# Post-traumatic Stress Disorder in School-age Children: A Nationwide Prospective Birth Cohort Study

**DOI:** 10.1007/s40653-024-00611-y

**Published:** 2024-02-22

**Authors:** Mogens Nygaard Christoffersen, Anne A. E. Thorup

**Affiliations:** 1 VIVE, The Danish Center for Social Science Research, Copenhagen, Denmark; 2 Faculty for Health and Medical Sciences, University of Copenhagen, Copenhagen, Denmark

**Keywords:** Childhood trauma, Community violence, Individual vulnerabilities, Life event history

## Abstract

Traumatic childhood events are some of the few identifiable and to some extent preventable causes of psychiatric illness. Children exposed to severely stressful events may react with post-traumatic stress disorder (PTSD) and this may impact their level of function in daily life, their future development and mental health. The traumatic stress model suggests that traumatic stress in the family, community violence, and other traumas are regarded as additive environmental factors that can outweigh protective compensatory factors and thus interact with individual vulnerabilities. This study is based on prospective panel data including the whole population of children born in Denmark from 1984 to 1994, who are followed from age 7 to age 18 (N = 679,000) in the window between 2001 and 2012. Risk factors for first-time diagnose with PTSD are analyzed by the discrete time log-odd model. We found a lifetime prevalence of 2.3% PTSD in school-age children (n = 15,636). In accordance with the model, indicators of traumatic stress in the family, family disintegration, community violence, and individual vulnerabilities predicted later diagnose with PTSD. Individual neurodevelopmental disorder – especially autism (adjusted Odds Ratio (OR 7.1) and ADHD (OR 10.7) – were predicative of PTSD. The results cooperated the traumatic stress model. Some results were inconsistent with the traumatic stress model e.g., parental substance abuse were associated with less than expected PTSD in school-age children when adjusted for other risk factors. This indicates that PTSD may be underestimated in these groups. PTSD diagnoses in administrative records underestimate the prevalence, systematically. Efforts to increase PTSD screening may allow for better management.

## Introduction

Traumatic childhood events are some of the few identified causes of psychiatric illness (Kerns et al., 2015). When children and adolescents are exposed to traumatic events considered as serious life stressors outside the range of normal human experience, the societal, interpersonal, and psychological consequences can be considerable (Keane & Barlow, 2004; Koss et al., 1991; Kulka et al., 1990; Toth et al., 2013). Children who are exposed to severely or continuous stressful events may react in a way that develops into post-traumatic stress disorder (PTSD), which can impact their level of function in daily life, and potentially their future development and mental health prognosis. Stressful events that can lead to PTSD in a child of school-age are defined as events that imply that the child is exposed to or witnessing a stressor that they perceive as threatening to their physical and/or psychological integrity of self (Blacker et al., 2019). PTSD is defined from a specific set of well described symptoms (e.g. flashbacks, nightmares, avoidance, memory lapses, emotional numbing and hypervigilance, DSM-5: 309.81 or WHO: F43.1) that persist for more than a month and impact the individual’s functioning (American Psychiatric Association, 2013; Kerns et al., 2015) and is related to a specific event or a series of events. If the reactions last less than a month, it is called acute stress disorder, and if it lasts longer than 6 months, it is called chronic PTSD.

Three primary types of trauma are often mentioned in relation to the development of PTSD (Foy et al., 1996): *community violence* (e.g. war, inner city gang-related violence), *accidents* (e.g. transportation accidents, natural disasters), and *family or individual trauma* (e.g. interpersonal trauma, child abuse and neglect).

This study investigates how constraining factors influence PTSD symptoms in school-age children. In general, the risk of developing PTSD is known to comprise a combination of early psychosocial factors, adverse environmental factors, and genetic and biological factors that through complex interactions may lead to PTSD. PTSD is rather common as a comorbid condition to neurodevelopmental disorders such as autism and attention-deficit hyperactivity disorder (ADHD) (Green & Ben-Sasson, 2010; Tannock, 2009) and is also common in combination with for example depression, eating disorders and psychosis.

### Lifetime Prevalence

Previous epidemiological studies have found a lifetime prevalence of PTSD of 6% in a community sample (US) of older adolescents using the NIMH Diagnostic Interview Schedule (Giaconia et al., 1995), lifetime prevalence of PTSD of 4.5% for 13- to 17-year-olds in the national (USA) comorbidity survey (Kessler et al., 2012), and lifetime prevalence of PTSD of 8.1% in adolescents under the age of 18 in the U.S. population (Kilpatrick & Saunders, 1999). Accordingly, the estimated lifetime prevalence of PTSD was found to be 6.6% in a previous study of ages 15–24 in a national comorbidity survey, USA (Kessler et al., 1995).

Variation in definitions and data sources causes problems when comparing results from studies on stress and trauma reactions in children and has thus hampered the study of the natural history and etiology and reactions to traumatic childhood events. The relatively small sample sizes make it difficult to study the effect of rare risk factors such as victims of physical and sexual abuse, war or harassment (Dyregrov & Yule, 2006). Many previous studies have used self-reported questionnaires instead of a clinical PTSD diagnosis (Tortella-Feliu et al., 2019). Therefore, there is a need for methodologically sound studies that pursue general population estimates and the etiology of clinically diagnosed PTSD in school-aged children.

### Previous Studies on the Etiology of PTSD

**Fig. 1:**
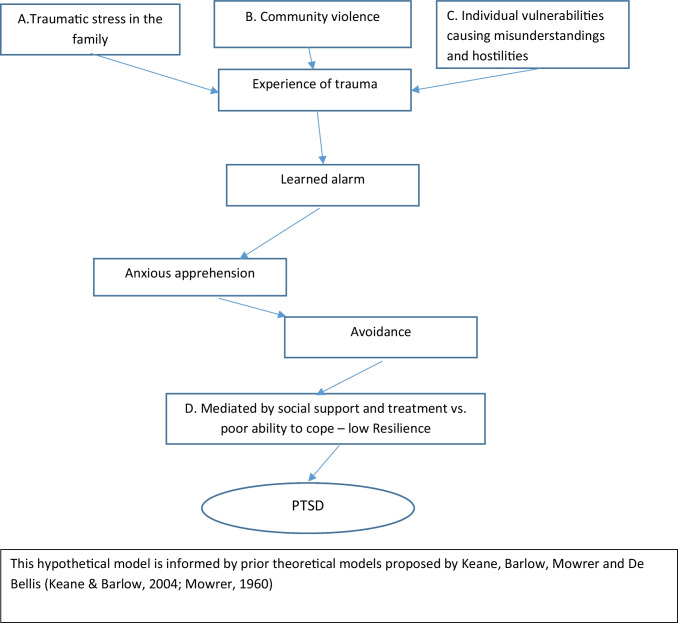
A model of the etiology of PTSD

The present study is inspired by Kean and Barlow’s learning theory (see Fig. 1) and other traumatic stress models (Yehuda et al., 2015) which posits that anxiety learned through associations and individuals will do whatever is necessary to escape and avoid cues that stimulate these aversive emotional memories. After a traumatic event, alarm signals emerge from one special chain of events and occur during exposure to situations that resemble the traumatic event. The anxiety is moderated by adequate coping skills and availability of social support and treatment. Identification of the precipitating event or proximal cause of PTSD is relatively simple, based on the theoretical descriptions of this form of anxiety (Keane & Barlow, 2004). Many factors affect the development of PTSD: genetic vulnerabilities; frequency and intensity of trauma; developmental stage at which trauma occurs; and comorbid psychiatric and substance use disorders (Blacker et al., 2019).

In the following, we present factors that previous studies have shown to be pertinent to consider when investigating the etiology of PTSD and other anxiety disorders in children. We use the findings of this previous research to inform our study design.

PTSD in children have been associated with negative life events in childhood such as having experienced maltreatment, e.g. sexual abuse, physical maltreatment, domestic violence, peer victimization or community violence (De Bellis, 2001; Luthar & Goldstein, 2004; Mynard et al., 2000). Such childhood experiences have also been associated with increased risk of psychopathology, including psychosis later in life (Varese et al., 2012). Interpersonal trauma, in cases where the perpetrator is a family member, has also been shown to lead to higher rates of PTSD in children and adolescents and erodes social support (Alisic et al., 2014; McLeer et al., 1992). However, not all children exposed to maltreatment or other traumas develop PTSD symptoms. This is believed to be due to compensatory factors in the environment and resilience factors in the individual (Alisic et al., 2014).

Longitudinal studies of continuous or chronic maltreatment show that chronicity must be taken into consideration to clarify the impact of maltreatment on the individual’s further mental health (Ethier et al., 2004). There is a need for nationwide studies investigating risk factors’ relationship to psychiatric disorders such as PTSD (Keane & Barlow, 2004).

(A) Traumatic stress in the family.
  The traumatic stress model tends to explain PTSD, the child’s psychosocial maladjustment, as a consequence of traumatic stress during the formative years. Such traumatic stress can be caused by child maltreatment, which is a severe form of interpersonal trauma to which the child is exposed on a daily basis or in periods (De Bellis, 2001; Ethier et al., 2004). Children exposed to domestic violence often meet diagnostic criteria for PTSD (Evans et al., 2008; Kilpatrick & Williams, 1998; Koolick et al., 2016; Levendosky et al., 2013).
  1. Parental mental health.
    Parents’ mental health problems can result in inadequate parenting and can contribute to cognitive difficulties and executive dysfunction in relation to caregiving. Children’s reactions to traumatic events are to some degree explained by the mother’s own mental health rather than exposure to the event (Dyregrov & Yule, 2006; McFarlane et al., 1987), and children will refrain from discussing a traumatic event if they register that doing so upsets their parents (Dyregrov & Yule, 2006).
    De Bellis proposes that child maltreatment and associated factors of parental substance abuse and family violence may be the result of “untreated” psychopathology, for example, severe mental illness, severe substance-abuse-dependence, antisocial personality disorder, or violent and criminal behaviors (De Bellis, 2001). Long-lasting parental mental disorder is a key variable found in studies of PTSD in children. Childhood PTSD has also been found to be correlated with both children’s psychiatric symptoms and trauma-related symptoms in their parents (Foy et al., 1996).
(B) Living in a disadvantaged area.
  In some inner-city areas in the USA, children have a high risk of being exposed to community violence. Parents and other adults can provide valuable support but are limited in how much they can offset the effects (Luthar & Goldstein, 2004). Therefore, the methodologically challenge is to disentangle community violence from restrains in the family (Fig. 1).
(C) Individual vulnerability.
  Biological stress system’s regulation will be based on the nature of the stressors, their frequencies, the chronicity of the stressors, and individual differences, especially genetic vulnerabilities (De Bellis, 2001). For example, individual genetic and biologic developmental deviances that lead to neurodevelopmental disorders such as ADHD and autism spectrum disorder (ASD) can lead to more frequent situations with stress or conflicts with peers and family members, the immediate neighborhood and other social settings. For example, neurodevelopmental disorders in schoolchildren like ADHD, ASD or mild mental retardation are associated with an increased risk of social misunderstandings and environmental conflicts. Social isolation, family stress and poor communication skills are examples for factors that increase the risk of maltreatment in children with disabilities (Sullivan & Knutson, 2000). Therefore, children with ASD and ADHD are at an increased risk of experiencing trauma-related symptoms due to daily stressors such as social confusion, peer rejection, punishment, being reprimanded, difficulties coping with changes, and due to their difficulties in regulating emotions and coping with stress (Kerns et al., 2015). A child with e.g. ADHD will often lack social support from friends (Bagwell et al., 2001; Barkley, 2000; Stroes et al., 2003).
  Children with ASD have profound and innate social cognitive disabilities which includes impaired communication and social interaction, and which makes it difficult for them to deal with social situations and relations and emotional regulation. Some children with ASD describe that they live in an environment in which they are unable to make themselves understood (Peeters & Gillberg, 1999). These ‘invisible’ disabilities may cause misunderstandings in social situations, and can lead to hostilities, bullying or isolation from peers and others (Petersilia, 2000). Further, these children lack adequate coping skills and have less social support from friends. Children with ADHD or ASD are more often victims of sexual and violent crime according to police records (Christoffersen, 2019, 2020). Aggressiveness and hostilities from the environment may cause anxiety in the children and adolescents, who find their environment incomprehensible.
  1. Social support as a mediating factor for creating resilience.
    Most individuals are able to adapt to normal life after a traumatic event due to resilience-giving factors, while a minority develop PTSD (Blacker et al., 2019). Resilience is defined as the ability to adapt successfully in the face of adversity, trauma, or significant threat (Horn & Feder, 2018) and maintain or reestablish a level of good function after the traumatic event. Social support from family members or friends is an example of a protective or buffering factor, while decreased social support could be caused by the parent’s own anxiety, depression or any other mental illness, high levels of family conflicts or family separation. Lack of social support to build resilience in the formative years and the timing, intensity, and chronicity of stress and trauma exposure can result in the development of PTSD and other stress-related disorders in children (Horn & Feder, 2018).
  2. Early pre-adoptive child maltreatment.
    Many adopted children have been exposed to some kind of adversity prior to being adopted, although most adopted children show psychological adjustment within the normative range (Bimmel et al., 2003; Juffer & Van Ijzendoorn, 2005). One study reported that adopted children had fewer behavioral problems than their non-adopted siblings or peers who stayed behind in orphanages or foster homes (Christoffersen, 2012), while another has shown that adopted children who experienced severe deprivation in their first months or years before adoption are at an increased risk of displaying both externalizing and internalizing behavioral problems (Hornfeck et al., 2019; Sonuga-Barke et al., 2017). Such problems persist during adolescence, and adopted adolescents report a higher amount of psychological distress and emotional problems than their non-adopted peers growing up with their biological parents (Askeland et al., 2017; Hornfeck et al., 2019; Olsen, 2017). There is no evidence that early adoption is a trauma for the individual, while ongoing negative life circumstances can undermine the person’s self-esteem and sense of well-being (Brodzinsky et al., 2022). These findings imply that it is appropriate to look more closely at PTSD in adopted children, although family-related factors such as social support, parental warmth, sensitive and positive parenting in adopted families predict positive outcomes for adopted children (Hornfeck et al., 2019).

## Child Substance Use and PTSD

Alcohol consumption above sensible drinking limits increases the vulnerability and the risk of PTSD in the adult population (Flensborg-Madsen et al., 2011). It is unclear if cannabis use increases the risk of developing long-lasting anxiety disorders (Crippa et al., 2009). Alcohol and various forms of illicit substance abuse could also be seen as “self-medication” to ameliorate stress and any kind of anxiety symptoms including PTSD in adolescents exposed to child maltreatment (Kosiba et al., 2019).

We hypothesize that following factors may increase their risk of encountering traumatic events and of developing clinically diagnosed PTSD (Fig. 1):

- A.1 Indicators of potential traumatic stress in the family (e.g. parental mental health problems, parental substance abuse, parental anxiety, long-term unemployment)
- A.2 Traumatic stress in the family (e.g. child maltreatment, domestic violence)
- B. Community violence (e.g. living in a disadvantaged area, victim of violence or sexual assault)
- C. Individual vulnerabilities (e.g. ADHD, ASD, mental retardation)
- D. Social support vs poor ability to cope (e.g. family separation, child in (public) care, child adopted)

The majority of trauma survivors do not develop PTSD (Yehuda et al., 2015). The study may help solve the conundrum of why most individuals are resilient when experiencing a sort of traumatic event, while only a minority develop PTSD (Blacker et al., 2019; Kessler et al., 1994).

## Methods

### Research Design

This study is prospective and based on longitudinal data from Denmark including the whole population sample, which provides information about the chronological sequence of potential causes before first-time registration of PTSD.

### Study Population

The study focused on national birth cohorts that included individuals born between 1984 and 1994, aged 7 to 18, resulting in a total of 679,000 individuals. The primary objective was to longitudinally track these individuals from childhood (age 7) through late adolescence (age 18) until their initial PTSD diagnosis was confirmed within a psychiatric ward. The dataset comprehensively covered various aspects, including personal vulnerabilities, instances of familial traumatic stress, family dynamics, exposure to community violence, and demographic factors. Data extending back to 1980 was available for both the cohort children and their parents. However, specific data regarding PTSD diagnoses was limited to years after 1993. Additionally, records concerning victims of sexual or violent assault were confined to years after 2001, sourced from police records. This prompted the study's focused investigation solemnly on the person-years during the period of 2001–2012 to encompass potential victims.

### Measures

#### PTSD (Dependent Variable) and Other Anxiety Disorders

The study is based on medical providers’ identification, assessments and diagnoses of anxiety disorders in a hospital ward, as they are typically the initial point of contact for PTSD cases in school-age children (Ramsdell et al., 2015). Cases were identified through the hospital’s records using ICD-10 diagnoses (WHO, 1994).

#### Risk Factors (Covariates)

##### Indicators of Traumatic Stress in the Family

Risk factors of parental mental illness, parental suicidal behavior, parental substance abuse, parental violence, and parental long-term unemployment were all included in the traumatic stress model (see Table A2 in Appendix). Parental anxiety disorder according to mental health center or GPs (according to drug prescriptions) were included as a potential risk factor.

Child maltreatment was operationalized according to hospital ward assessment (ICD-10: T 74, Z 61, Y 07.1, Y 06.1) which included maltreatment syndromes, neglect or abandonment, physical abuse, sexual abuse, psychological abuse).

##### Indicators of Disintegrative Family

Table A3 in Appendix shows the selected indicators disintegrative family e.g. child in care, family separation or the child being adopted of adversity before being adopted as well as indicators of lack of social support from families indicated by family dissolution or being in public care.

##### Indicators of Community Violence

Living in a disadvantaged area is operationalized in Table A4 in the Appendix. Potential indicators of traumatic life events are involvement in violent crime. The child being a victim of violence or sexual assaults were also included as indicator of community violence.

##### Individual Vulnerabilities

Children’s neurodevelopmental disorders and other disabilities are considered individual vulnerabilities. The types of disability are based on a database mandated, compiled and maintained by Danish hospitals in accordance with the international statistical classification of diseases (ICD-10) and health-related problems (WHO, 1994). We classified disabilities into 14 main groups, which did not cover all disabilities (Table A5 in Appendix). The categories did not include disabilities that could be consequences of maltreatment such as internalizing disorders, depression, and other emotional disorders.

For the sake of our analysis, some disabilities that may affect the adolescent’s ability to communicate were grouped under speech disability (i.e. developmental disorders of speech and language), while other disabilities such as cerebral palsy was kept in a separate category.

Neurodevelopmental disorders like ASD, ADHD, mental retardation and dyslexia were diagnosed in mental health services, which all report their data to the Danish Psychiatric Nationwide Case Register. Cases of ADHD are also recorded if a child has received ADHD medication prescribed by medical practitioner or specialist (Table A5 in Appendix).

Potential demographic risk factors such as parental teenage-motherhood, non-Danish citizens, and gender are included (Table A6 in Appendix).

## Data Analysis

Our model allows that individuals can have multiple disabilities (Table A5 in Appendix). When reviewing the effect of a specific type of vulnerability in the regression analysis, the reference group would be the person years without that specific type of disability. The model allows for changing covariates and changing disability over time. The study takes advantage of analysis of covariance and multiple regression statistical analysis methods so that the interrelationship between several predicative variables and first-time PTSD can be examined simultaneously, as recommended by some researchers (Foy et al., 1996).

The data is analyzed by the discrete time-log-odds model (Allison, 1982). A discrete-time model treats each individual history as a set of independent observations. The shortest time interval possible is a calendar year. Individuals’ history is broken up into 12 sets of discrete time units (age 7 to 18 years). Each individual is observed until either an event occurs, or the observation is censored by reaching the age limit, because of death, or because the individual is lost to observation for other reasons, for example immigration. Consequently, individuals are excluded from the case group and controls after the first event.

The person years at risk were constructed for the total birth cohort in the window between 2001 and 2012. Pooling the non-censored years of all individuals, the person years provided the numbers at risk (N = 4,917,535). It has been shown that the maximum likelihood estimator can be obtained by treating all the time units for all individuals as though they were independent, when studying first-time events (Allison, 1982).

The model allows for changing covariates over time. Some variables, such as gender, may be constant over time, while others, such as living in a disadvantaged area, anxiety, may vary. In an event history, the observation time in an administrative register, police records, and hospital’s records may be different from the time of the event. When ADHD or Autism have been registered in the hospital records and medical records at e.g. the age of 17, the disorder have probably been active the years before. The 35 covariates are divided into three types for the purpose of this study. The Type I covariates are those that are taken to be indicative throughout the risk period, irrespective of when the covariate was notified, for example parental substance abuse or child’s diagnosis of autism or ADHD. Covariates of Type II, in contrast, identify the presence of that factor in the year prior to the event, for example parental long-term unemployment during a calendar year, or moving into a disadvantaged housing area, until moving out. Finally, the Type III covariates act on the following year and all the subsequent years when observed the first time, for example family separation or brain injury. Missing data’s potential impact on the results are then minimized. The model is sensitive to changes in environmental stresses e.g. parents health, parental long-term unemployment, exposure or witnessing violence.

Finally, for each age group (age = 8, 9,.., 17) a constant (dummy) is estimated. The proportional log-odds model allows for standardization relating to age. Maximum likelihood estimators for the regression models are then calculated on the basis of pooling all the person years and log-odds are presented.

The logistic regression function is written:

$$\mathrm{Log}\lbrack{\mathrm P}_{\mathrm{it}}/(1-{\mathrm P}_{\mathrm{it}})\rbrack=\alpha_{\mathrm t}+\varvec\beta^{\prime} \varvec {\mathrm x}_{\mathrm{it}}$$

P_it_ is the conditional probability that individual *i* has an event at age t, given that it has not already occurred to that individual. **α**_t_ are dummy variables being 1 when the individual reach the age *t*, otherwise 0. The explanatory variables x_it_ take on different value at different discrete age *t* (*t* = 8,…,18). The observation continues until age t_i_, at which point an event occurs or the observation is censored (Allison, 1982). Censoring means that the individual is not observed beyond the age *t*_*i*_*.*

### Statistics

We wish to describe the environmental situation for the children the year before first-time PTSD diagnosis in comparison with their contemporaries. The multivariate analyses included 35 risk variables (covariates) covering various aspects of the traumatic stress model. In order to evaluate the risk factors’ contribution to the number of persons diagnosed with PTSD, attributable fractions (AF) are calculated (Greenland, 1998). Risk factors were included into the final model stepwise, if they contributed with new information, given all the others risk factors.

Attributable fractions express the reduction in incidence of PTSD that would be achieved if the population had not been exposed at all compared with the current exposure pattern (Greenland. & Drescher, 1993). The estimated AF of a certain risk factor depends on two parameters only. One parameter is the strength of the risk factor measured by adjusted Odds Ratio (OR) or relative risk (RR). The other parameter is the current exposure of the risk factor in the population. The estimated AF is calculated solely on the basis on these two parameters (Levin, 1953; Woodward, 1999). Attributable fractions are only defined when OR and RR is more than 1.

The lifetime prevalence is estimated on the basis of the first-time incidence rate for each age-group t = 7, 8, 9, …, 18. We imagine that 100,000 individuals live through the ages 7 to 18 years with the estimated age specific probabilities of first-time PTSD.

## Results

### Test of the Trauma Stress Model

The lifetime prevalence of PTSD in school-age children diagnosed according to ICD-10 in a hospital ward was 2.3% (N = 15,636 following the eleven birth cohorts from seven to eighteen years).

Traumatic stress in the family was found to be predictive for PTSD in schoolchildren, after accounting for demographics (Table A5 in Appendix) and other environmental stressors and individual vulnerability (Table A2-A4 in Appendix). Traumatic stress in the family was indirectly indicated by parental suicidal behavior, parental mental retardation, parental substance abuse, parental anxiety disorders, or directly by parental violence and child maltreatment (Table A1 in Appendix). Parental suicidal behavior was found in 15.9% of cases, while parental PTSD was found among 10.8% of children diagnosed with PTSD. Other types of anxiety disorders in parents were found among 37.0% of children diagnosed with PTSD, and about the same amount was found regarding parental substance abuse (31.4%) and parental violence (26.3%), while child maltreatment was only recorded for 0.2% of children with PTSD (Table 1).

**Table 1:** Risk factors indicating potential traumatic stress in the family and PTSD

***Risk factor:***	**Type**	**% of**	**% of**	**PTSD**		
***The family***		**Controls**	**Cases**	**OR**	**95% CI**	**AF %**
Parental suicidal behavior	(I)	7.1	15.9	Ns	-	-
Parental mental retardation	(I)	3.1	8.1	1.07*	[1.00–1.15]	0.2
Parental PTSD	(III)	3.9	10.8	1.19***	[1.12–1.27]	0.7
Parental anxiety disorders	(III)	19.1	37.0	1.25***	[1.20–1.30]	4.6
Parental substance abuse	(I)	15.7	31.4	0.92	[0.88–0.97]	-
Parental unemployed > 21 weeks	(II)	6.9	6.9	0.84***	[0.79–0.90]	-
Parental violence	(III)	13.9	26.3	1.15***	[1.11–1.20]	2.1
Child maltreatment	(II)	0.02	0.22	1.89**	[1.32–2.72]	0.02

The mentioned traumatic stressors predicted PTSD in the children the following year, when all the risk factors were taken into account. The adjusted effect sizes were between OR 1.19 and 1.25 for parental PTSD and parental anxiety. The results indicate that if a child knows their parents have been involved in violence as victim or offender, the child is at an increased risk of being diagnosed with PTSD in school age the following year (OR: 1.15; CI 1.11–1.20). Child maltreatment that leads to hospitalization and professional assessment of the injury being willfully inflicted by other persons, was found to be at an increased risk of being diagnosed with PTSD the following year (OR: 1.89; CI 1.32–2.72). However, only few cases (0.22%) were detected in the hospital files (AF 0.02). Although significant, these indicators of family stressors explained only a minor proportion of the risk of PTSD in school-age children when all the known risk factors were taken into account (Table 1). About 4% of the cases of PTSD could be attributed to exposure to parental anxiety disorders (AF 4.55).

We found that children exposed to community violence are predicative of PTSD (Table 2). For example, a child being a victim of violence, or sexual assault according to police records were diagnosed with PTSD the following year (OR 1.45; CI 1.35–1.56; and OR 1.54; CI 1.40–1.68), respectively. PTSD was also found among adolescents convicted of a violent crime (OR 1.26; CI 1.10–1.44). The association between exposure to violence and sexual assaults is strong, but since exposure to these threats is rare, only relatively few cases of PTSD in the population could be explained by these severe constraints.

**Table 2:** Family disintegration and PTSD

***Indicators of risk factors:***	**Type**	**% of**	**% of**	**PTSD**		
		**Controls**	**Cases**	**OR**	**95% CI**	**AF %**
Child in (public) care	(II)	1.9	10.3	Ns	-	-
Child adopted	(I)	1.2	2.5	1.16*	[1.04–1.29]	0.19
Family separation	(III)	35.8	56.9	1.12***	[1.08–1.16]	4.11

Living in a disadvantaged area seemed to be a protective factor, when adjusted for the other risk factors (OR 0.83; CI 0.74–0.93).

Regarding family disintegration, we found that children approximately 2.5% of children with PTSD were adopted. Being adopted predicted later PTSD in school-age children (OR 1.16) while family separation showed a similar predictive effect size (OR 1.12; AF 4.1). About half of the children with PTSD (56.9%) had experienced family separation Table 3.

**Table 3:** Indicators of community violence and PTSD

***Risk factor for individual schoolchild:***	**Type**	**% of**	**% of**	**PTSD**		
		**Controls**	**Cases**	**OR**	**95% CI**	**AF %**
Charged of violence	(III)	0.3	1.7	1.26**	[1.10–1.44]	0.08
Victim of a sexual crime	(III)	0.7	3.6	1.54***	[1.40–1.68]	0.36
Victim of a violent crime	(III)	1.2	5.8	1.45***	[1.35–1.56]	0.56
Disadvantaged area	(II)	2.2	2.0	0.83**	[0.74–0.93]	-

Ns means Non-significant i.e. *p* > 0.05. Adjusted odds ratio. Type of dependency. Type I: Risk factor observed at time t also covers the years before and after the years under investigation. Type II: Exposed to risk factor at time t are also present at t + 1. Type III: Exposed to risk factor at time t, then the risk factor is also present at all the following years

*p < 0.01; **p < 0.001; ***p < 0.0001

**Fig. 2:**
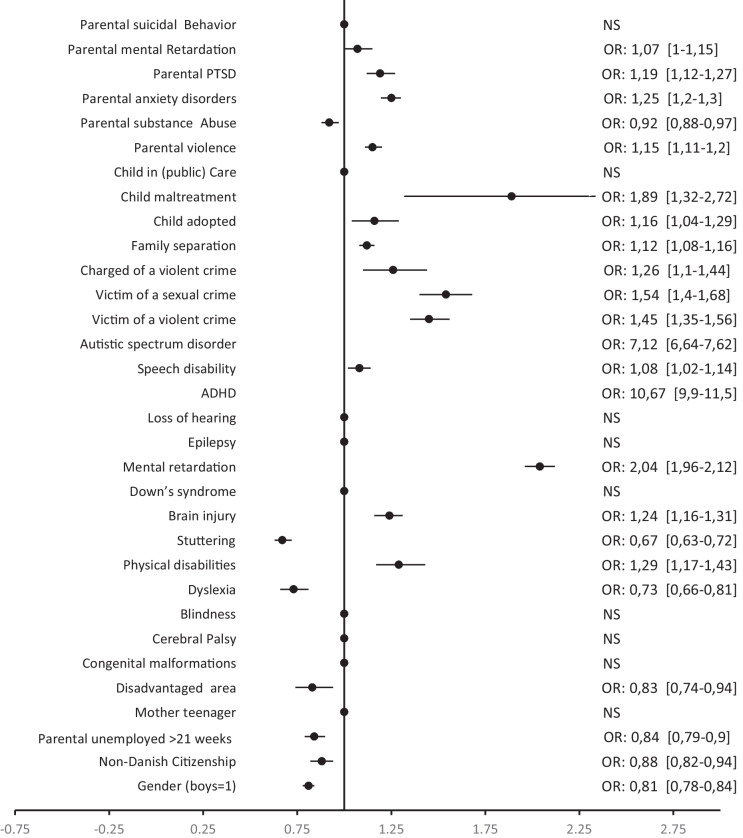
Forrest plot of subgroups, post-traumatic stress disorder, adjusted Odds Ratio (OR). [95% CI] Note: the plot of ADHD OR 10.69 95% CI [9.92-11.53] is not included in Fig. 2. Likewise, neither is ASD OR 7.14, 95% CI [6.67-7.65] included in the plot

The results in Table 4 show that individuals with neurodevelopmental disabilities such as ASD or ADHD have approximately seven- to ten-times higher risk of being diagnosed with PTSD, when other potential risk factors are taken into account (OR: 7.14–10.69; AF 28–44%) Fig. 2.

**Table 4:** Individual vulnerability (disability) and PTSD

***Risk factor:***	**Type**	**% of**	**% of**	**PTSD**		**AF**
**Disabilities:**		**controls**	**Cases**	**OR**	**95% CI**	**%**
Autistic spectrum disorder	(I)	6.4	83.7	7.14***	[6.67–7.65]	28.15
Speech disability	(III)	7.9	23.2	1.08*	[1.02–1.14]	0.62
ADHD	(I)	8.2	87.4	10.69***	[9.92–11.53]	44.34
Loss of hearing	(III)	1.1	1.8	Ns	-	-
Epilepsy	(I)	1.5	3.6	Ns		-
Mental retardation	(I)	4.6	64.8	2.02***	[1.94–2.10]	4.44
Down’s syndrome	(I)	-	-	Ns	-	-
Brain injury	(III)	4.6	8.4	1.24***	[1.16–1.31]	1.09
Stuttering	(III)	1.3	13.6	0.67***	[0.63–0.72]	-
Physical disabilities	(III)	1.3	2.7	1.29***	[1.17–1.43]	0.38
Dyslexia	(III)	0.3	2.7	0.73	[0.66–0.81]	-
Blindness	(I)	0.1	0.2	Ns	-	-
Cerebral Palsy	(III)	0.3	0.5	Ns	-	-
Congenital malformations	(I)	0.8	1.2	Ns	-	-

Mental retardation increased the risk by about 100% (OR for PTSD: 2.02; AF 4.4). Children with physical disabilities were also found to have elevated risk of PTSD (OR was 1.29), while rare disabilities such as blindness, loss of hearing or congenital malformation were not found to be significantly associated with increased risk of PTSD.

Surprisingly, we found children with stuttering or dyslexia to have less risk of being diagnosed with PTSD compared to school-age children without these disabilities (OR for PTSD was: 0.67, and 0.73, respectively).

School-age girls had a higher risk of PTSD compared to school-age boys, when adjusted for other known risk factors for girls (OR: 1.24), corresponding to OR: 0.81 for boys.

Risk factors of PTSD are being a victim of a violent crime or sexual crime, parental violence, child being battered or neglected (i.e. child maltreatment), or the child being convicted of violence. Contrary to expectations, parental substance abuse, parental long-term unemployment, living in a disadvantaged area and non-Danish citizenship were found to be associated with lower levels of PTSD, when adjusted for the other known risk factors.

## Discussion

The aim of this study was to contribute to our understanding of the etiology and risk factor of PTSD in children and adolescents by using Danish register data to evaluate associations between childhood traumatic events and later diagnosis of PTSD in school-age children nationwide. We found a lifetime estimation of PTSD in school-age children in the range of 2.3%. The PTSD diagnosis was made in psychiatric wards, and it is likely that this result is underestimating the amount of PTSD in school-age children. We assume that less severe cases were not be included in the present study, since they were treated in the primary sector or not treated at all.

We found an increased risk of PTSD among children diagnosed with neurodevelopmental disorders such as ASD, ADHD, and mental retardation. Children exposed to domestic violence, child maltreatment and children who were adopted showed an increased risk of PTSD. These estimates are most likely in the lower end due to hidden data on anxiety disorders, and the underreporting or lack of reporting in the registers. The results have similarities with previous studies, but the results also indicate structural divergences.

Previous studies of PTSD based on students’ self-reports estimated lifetime prevalence of 6% in a small community sample of older adolescents (N = 384), while a small Danish study (N = 390) estimated a 9% lifetime prevalence of PTSD (Elklit, 2002; Giaconia et al., 1995).

The results of the present study should be understood in the light of previous findings that child maltreatment in a broad understanding is a preventable contributor to later development of mental illnesses in adolescence (De Bellis, 2001). Other studies have found that when rescued from extremely abusive environments, some profoundly maltreated children are capable of catching-up on growth, including normalization of cognitive function (De Bellis, 2001; Koluchová, 1976; Money et al., 1983; Sonuga-Barke et al., 2017).

Contrary to what we expected from previous findings parental substance abuse, parental long-term unemployment, living in a disadvantaged area, and non-Danish citizenship were all associated with a significantly reduction in the risk of PTSD in their children. We assume that children in these families have a least the same risk of PTSD as their contemporaries, but depression, PTSD, and anxiety are relatively more seldom screened, assessed, and diagnosed in these group of children. An explanation could be that children with PTSD could spend a quiet life without being assessed. The results indicate that the outcome PTSD is assessed more thoroughly in some groups than others. This type of statistical problem found in hospital records are also known as Berkson’s bias (Berkson, 2014; Westreich, 2012).

We found a surprisingly high prevalence of ASD among school-age children diagnosed with PTSD. This finding was surprising, as it was even higher than the high prevalence of anxiety and other mental health problems that previous research has already documented in children and adolescents with ASD, when compared with community-based populations. Differentiating between clinical presentations of only anxiety and anxiety from underlying ASD and determining when these two are co-morbid diagnoses, can be difficult in real life, since symptoms of anxiety are very common both in children in general and in those who have ASD (MacNeil et al., 2009).

Differentiating between PTSD and the core characteristics of ASD is in most cases easily done, but PTSD and ASD can present in many forms. However, we conclude that the high prevalence of PTSD among school-age children with ASD must be understood both as a co-morbid phenomenon and as a secondary condition that develops more easily due to a low threshold for PTSD in school-age children with ASD or ADHD because of their problems with understanding and interpreting all kinds of stimuli from their surroundings.

The occurrence of ASD and ADHD increases the likelihood of being assessed in a hospital ward. The quality of assessment and treatment of children with ASD or ADHD may confirm the presence of PTSD. The strong association between PTSD and ASD or ADHD may partly be a result of the mentioned Berkson’s bias.

PTSD can be understood as a natural or evolutional protection mechanism that serves as a safeguard against experienced threats to well-being or survival. A child with ASD perceives the social environment as difficult to predict and understand and may thus often also experience stress, anxiety or lack of control. The child will therefore be at an increased risk of experiencing trauma, which can lead to development of PTSD, also just on the basis of situations that others would perceive as simple routine activities or just random events that can happen (e.g. the death of a pet). Furthermore, misunderstandings and violent behavior due to poor emotion regulation, poor emotional expression and limited ability for introspection and reflection can result in conflicts and intrusive sanctions from surroundings, which may cause trauma and later PTSD in children with ASD (Barlow, 2004).

Child maltreatment and other traumatic life events affect school-age girls to the same degree as school-age boys. As expected, we found that school-age girls have a higher risk of PTSD compared to school-age boys, which may be related to differences in type of exposure (Alisic et al., 2014). Boys are more often exposed to violent crime, while girls are more often exposed to sexual assaults. Previous studies have found that exposure to interpersonal violence, for example sexual and physical assaults, is associated with increased risk of PTSD (Kilpatrick et al., 1992; Resnick et al., 1993; Wolfe et al., 1994). Other studies confirm that school-age girls have higher rates of internalization of disorders, and self-blaming (Alisic et al., 2014; Tolin & Foa, 2008).

Some risk factors are closely associated to PTSD both as a consequence and as a cause. The present data cannot disentangle these factors. Suicidal behavior, drug abuse and alcohol abuse can be a consequence of PTSD. These three risk factors are therefore not included in the previous adjusted model when all other risk factors’ effect sizes (odds ratios) are estimated (Tables 1–5).

**Table 5:** Demographic variables and PTSD

***Demographic risk factor:***	**Type**	**% of**	**% of**	**PTSD**		**AF**
		**Controls**	**cases**	**OR**	**95% CI**	**%**
Mother teenager	(I)	1.9	3.2	Ns	-	-
Non-Danish citizenship	(III)	8.5	5.7	0.88***	[0.82–0.94]	-
Gender (boys = 1)	(I)	51.3	44.0	0.81***	[0.78–0.84]	-

Previous studies of the etiology of anxiety disorders have often been based on retrospective measures of potential risk factors, but such studies may be hampered by recall bias. Some studies of the etiology of PTSD are based on small non-probability samples, often have a relatively large proportion of non-responses, and failure to collect prospective data. The lack of relevant comparison groups may compromise the ability to evaluate the etiology of anxiety (Sicotte et al., 2018).

To obtain data for all important outcomes of severe constraints on school-age children, it is necessary to examine long-term follow up studies based on observational data. Non-randomized population studies might be the only way to study effects of, for example, maltreatment, environmental reactions to individual vulnerabilities and PTSD. As previous studies repeatedly report relatively rare but significant associations with PTSD in school-age children, it is essential to identify from an early age the previous risk factors and describe their relative effect on development of PTSD. When small potential risk factors mount up in the patchwork of causes, there is a crucial need for large-scale studies.

We found that stuttering or dyslexia seemed to be protective against developing PTSD, since school-age children with these disabilities had a lesser risk of being diagnosed with PTSD compared with school-age children without these disabilities, when all known confounders were accounted for. An explanation for this could be that children struggling dyslexia and children who stutter are offered help from speech therapists and that free education is accessible to these children along with advice to parents and schoolteachers. We speculate if children with dyslexia or stuttering may experience effective social support from close surroundings. Stuttering and dyslexia are disabilities that impact a smaller part of life than ADHD or ASD, which are characterized as causing more general impairments in daily life functioning.

Our results were consistent with the described traumatic stress model. Children exposed to maltreatment or other kinds of traumatic stress (e.g. traumatic stress in the family, pre-adoptive deprivation, sexual abuse, violence in the family, community violence) will almost always react with fear, re-experiencing of the trauma and will be likely to avoid stimuli associated with the trauma. Interpersonal trauma where the perpetrator is a family member increases the risk of PTSD and erodes social support, since the child lives with the person who represents the trauma. Parents’ own anxiety symptoms or other mental health problems may reduce their capacity to support children. The lack of available social support for resilience and the exposure to traumatic stress were associated with diagnosed PTSD in school-children.

Post-traumatic stress disorder impacts the individual’s daily level of function, and their future development and mental health. Post-traumatic stress disorder is furthermore associated with increased risk of suicidal behavior, drug abuse and alcohol abuse (De Bellis, 2001).

### Practical Implications

PTSD in children are often found in individuals with neurodevelopmental disorders and almost always affect daily functioning. PTSD may be overlooked in some groups of children, and non-treated, it may have long-lasting impact on their behavior and their social and emotional well-being. This makes PTSD a public health problem of concern in relation to the most vulnerable school-age children. The implementation of early interventions to identify school-age children who are at an increased risk of trauma and secondary stress reactions could be one way of reducing the prevalence of PTSD, societal cost, and long-term disability (Ramsdell et al., 2015).

Future studies should focus on how the consequences of traumatic experiences may lead to stress disorders including PTSD in children and adolescents. Our findings underline the importance of understanding the traumatic reactions that might develop in response to traumatic family stressors such as child maltreatment, family violence, hostile and putative environment, lack of social support, insufficient assess to treatment in combination with individual vulnerabilities.

Cognitive Behavioral Therapy (CBT) is recommended as first choice treatment of schoolchildren (age 6 to 17 years old) for social phobia (ICD-10: F40.1; DSM-5: 300.23), separation anxiety (ICD-10: F93.0; DSM-5 309.21) or generalized anxiety (ICD-10: F41.1; DSM-5: 300.02). The Danish Health Authority suggests in the National Clinical Guideline for the treatment of obsessive-compulsive disorder (OCD) that manualized family-based CBT and manualized CBT may both be considered for treatment of children and adolescents with OCD (Arendt et al., 2016; NICE, 2013; Rosenberg et al., 2007). The administrative registers or medical records do not include information about CBT.

Treatments of PTSD in school-age children

Approx. 15% of children who experience one severe trauma or several severe traumas develop PTSD. The recommended treatment is trauma-focused cognitive therapy (de Arellano et al., 2014), which shows good evidence for improvement on various outcome measures. However, trauma-focused cognitive therapy demands special training and is, to the best of our knowledge, far from implemented in Denmark as a standardized treatment for children experiencing traumatic events.

Psychopharmacological agents such as selective serotonin reuptake inhibitor (SSRI) and serotonin noradrenaline reuptake inhibitor (SNRI) are not recommended for the treatment of PTSD among children and adolescents (Arendt et al., 2016; NICE, 2013; Rosenberg et al., 2007). Only few randomized controlled trials have been conducted on the use of psychopharmacological agents for children following traumatic exposure, and a lack of sufficient documentation for the effects of psychopharmacological agents leads to researchers instead recommending CBT methods to reduce anxiety symptoms (Arendt et al., 2016; Dyregrov & Yule, 2006; NICE, 2013; Rosenberg et al., 2007).

### Ethical Problems

No previous nationwide prospective study has attempted to handle the many technical, methodological and ethical issues involved in investigating factors such as domestic violence, child maltreatment, sexual abuse and other kinds of traumatic events in the lives of children and these factors’ relationship to PTSD (Keane & Barlow, 2004). Ethics Committee/Institutional Review Board approval for the research was not obtained for register studies in Denmark. Instead data were accessed by approval from the Danish Data Protection Agency, and data were analyzed in an anonymous form via access to the database at Statistics Denmark. Ethical problems have been tackled by encryption and anonymity must be strictly adhered to in accordance with these principles.

### Limits of the Study

The method we used will most likely underestimate the lifetime prevalence of PTSD by age 18 years. The present study is solemnly based on hospitals’ records. Studies based on administrative records of diagnosed PTSD e.g. electronic health records do not capture all cases of PTSD due to underdiagnoses as well as underreporting (Schein et al., 2021). PTSD in children may be underdiagnosed in routine clinical practice, and therefore seldom referred to a psychiatric ward.

PTSD is undiagnosed in part due to a lack of awareness in the general population, stigma, and barriers to health care access (Schein et al., 2021). Children and their parents may not be aware of their symptoms or may be unwilling to disclosure the full scope of their children’s symptoms due to concern of stigmatization (Schein et al., 2021). The present study find accordingly, that some vulnerable groups may be underdiagnosed. We find that PTSD is underdiagnosed in some groups probably because they are particularly affected by barriers to health care access e.g. families with parental substance abuse, parental long-term unemployment, living in a disadvantaged area, and non-Danish citizenship.

We find the prevalence to be 2.3% while comprehensive studies find the lifetime prevalence to be approximately 6% (Giaconia et al., 1995; Rosenberg & Chiriboga, 2020). These discrepancies clearly indicate that diagnoses and treatment of PTSD in adolescence is incomplete and we fear that consequences are lack of social support, insufficient assess to treatment in combination with individual vulnerabilities.

The results indicate that children with ASD and children with ADHD have a five- to eight-times’ higher risk of being diagnosed with PTSD, when other potential constraints were taken into account. We assume that these figures overestimate the association. Post-traumatic stress disorder may in some cases only be reported in connection with investigating a child’s decreasing function or increasing of symptoms of ADHD or ASD. We have reason to believe that many children with PTSD do not receive sufficient treatment from psychiatric wards, municipal early interventions or GPs for the disorder, which may become chronic.

One drawback to the present study is that the data only provide information on diagnoses on personal vulnerabilities and lack information on existing and received effective treatment, and received social support from family and significant others. We use family disintegration and traumatic stress in the family as indicators of lack of social support. There is a need for further studies to understand the possible positive impact of social support and to adapt treatment strategies to counteract adverse outcomes.

A further limitation of this study is its inability to assess the impact of CBT, which is recommended as the first-choice treatment of schoolchildren with PTSD. The same limitation applies to trauma-focused cognitive-behavioral therapy, which has demonstrated positive outcomes in reducing symptoms of PTSD (de Arellano et al., 2014). Individual-level information on these measures are not made available for the use of research in the Danish administrative databases, police records, and journal of hospital records. Prospective longitudinal studies on large probability samples offer the best way to study predictors of environmental stressors causing trauma and possible PTSD in school-age children. The present study is based on a large sample with comprehensive information about potential risk factors for all individuals. This allows for disentangling of the predictors’ influence on the risk of developing PTSD. Unknown potential risk factors are the Achilles’ heel of the strategy. The administrative data system, police records, or hospital records gives little or no knowledge of individual measures and local initiatives taken in the primary sector to address potential traumatic life events and anxiety disorders in school-age children.

Data records concerning victims of sexual or violent assault were confined to years after 2001. This will result in incomplete representation of young people age 7 to 12, especially children born before 1990, while incidents against children born before 1990 will be exclusive included for the older age groups. We have no knowledge of the amount of underreporting incidents and variations in reporting practices across regions.

The study method has some inherent limitations because highly predicative traumatic events are rare events in the population, and many potentially traumatic events were not included in the data. Consequently, we must assume that only a relatively small part of PTSD cases in school-age children could be explained by the presented traumatic stress model.

New Danish studies have shown that the prevalence of some of the potential risk factors of PTSD in school-age children has increased during the last 10 years (2012 to 2022). Diagnoses such as ADHD/ADD and autism spectrum disorder (ASD) have increased since 1994 and also during 2012 to 2022. An explanation is that psychosocial support and tracing of vulnerable groups in general has increased during the last decades. Early support to the children and their families could reflect the increased numbers and therefore lessen the load and the risk of PTSD. On the other hand, in connection with the diagnosis of ADHD/ADD, ASD, the investigation could rule out or confirm the presence of the PTSD, depression, anxiety and other diseases. After 2012 changes to the diagnostic criteria for post-traumatic stress disorder (PTSD) for ICD-11 have been proposed and adopted with a more focused approach in order to identify the symptoms unique to the PTSD construct and avoiding symptoms that overlap with other disorders such as depression (O'Donnell et al., 2014). This would substantially reduce the number of individuals with the disorder (Wisco et al., 2016).

We are worried that it can be difficult to fully implement the changes to the diagnostic criteria for PTSD in adolescents because parents tend to underreport depressive symptoms of their offspring (Eg et al., 2018), and other studies have found that the majority of adolescents with MDD did not receive disorder specific treatment, and this discrepancy was greatest for minority youth (Avenevoli et al., 2015). Further research can show whether these developments is followed by an increase in PTSD incidence among school-age children during the period 2012–2022.

### Future Directions

It is estimated that one third of individuals who experience a severe traumatic event will develop PTSD (Tortella-Feliu et al., 2019). Key psychosocial factors associated with ability to adapt successfully in the face of adversity include the availability of social support for resilience across the lifespan (Horn & Feder, 2018). Interventions targeting adolescent’s social environment both at home and at school may be beneficial (Daniunaite et al., 2021). Future research should focus on resilience and the prevention and treatment of PTSD, for example by implementing easily accessible trauma-focused cognitive-behavioral therapy for children and adolescents in close relation to the traumatic event(s) (de Arellano et al., 2014). Dissemination of knowledge about the impact of trauma for mental health, the risk of developing PTSD and the ‘low hanging fruits’ of the intervention possibilities is also widely needed.

PTSD diagnose in administrative records based on hospital wards underestimated the prevalence of PTSD in adolescence. We are unaware of the extent of the missing data. Recent nationally representative estimates are lacking. Schein and colleagues conclude that efforts to increase of PTSD screening and improve awareness may allow for better management of PTSD (Schein et al., 2021).

Another key psychosocial factor is individual vulnerabilities (e.g. ADHD, ASD) that cause misunderstandings, hostilities or just negative feedback and experiences from a person’s surroundings. The positive role of psychoeducation and other social and educational interventions in children with neurodevelopmental disorders has been corroborated by a systematic review (Montoya et al., 2011). Further research is needed to investigate how psychoeducation can be used to change the psychosocial environmental conditions of at-risk children.

## Appendix

**Table A0:** PTSD (dependent variable) and other anxiety disorders

***Outcome factor:***	***Definition***
PTSD	*Post-Traumatic Stress Disorder ICD-10: F43.1,* diagnosed in a psychiatric ward according to the Danish Psychiatric Nationwide Case Register
Other Anxiety	*Other Anxiety disorders ICD-10: F40-F42.* Diagnosed with anxiety disorder in a psychiatric ward according to the Danish Psychiatric Nationwide Case Register
Pharmacological drugs	Anxiety disorders receiving drug treatment with one of the following psychopharmaca: N05BA12 Alprazolam, N0 6AB03 Fluoxetin, N06AB05 Paroxetin, N06AB06 Sertralin, N06AB10 Escitalopram, N06AX11 Mirtazapin, N06AX16 Venlafaxin, N03AE01 Clonazepam, N03AX16 Pregabalin. (Arendt et al., 2016; NICE, 2013)

The dependent variable includes only “PTSD” F43.0-F43.9.

**Table A1:** Risk factors indication potential traumatic stress in the family

***Risk factor:***	**Family vulnerability**
Parental suicidal behavior	Parents’ suicide attempts according to the National Patient Register and the Danish Psychiatric Nationwide Case Register, or suicide according to the Causes of Death Register. Intentional self-harm according to hospitals admissions is also included (Christoffersen et al., 2003a)
Parental mental retardation	Mental retardation ICD-10:F70-F79
Parental inpatient mental illness	One or both parents admitted to a psychiatric ward according to the Danish Psychiatric Nationwide Case Register
Parental PTSD	*Post-Traumatic Stress Disorder ICD-10: F43.1,* One or both parents diagnosed in a psychiatric ward according to the Danish Psychiatric Nationwide Case Register
Parental anxiety disorders	*Anxiety disorders ICD-10: F40-F42.* One or both parents diagnosed with anxiety disorder in a psychiatric ward according to the Danish Psychiatric Nationwide Case Register, diagnosed in a psychiatric ward or receiving drugs treatment with one of the following psychopharmaca: N05BA12 Alprazolam, N0 6AB03 Fluoxetin, N06AB05 Paroxetin, N06AB06 Sertralin, N06AB10 Escitalopram, N06AX11 Mirtazapin, N06AX16 Venlafaxin, N03AE01 Clonazepam, N03AX16 Pregabalin. (Arendt et al., 2016; NICE, 2013)
Parental substance abuse	Alcohol abuse or drug abuse (see below)
Parental alcohol abuse	According to hospital admissions the following diagnoses were expected to be associated with long-term alcohol abuse: Alcoholic psychosis, alcoholism, esophageal varices, cirrhosis of liver (alcoholic), chronic pancreatitis (alcoholic), delirium, accidental poisoning by alcohol. Mental and behavior disorder due to use of alcohol also included (Christoffersen & Soothill, 2003)
Parental drug abuse	Addiction or poisoning by drugs according to hospitals admissions. Mental and behavioral disorder due to use of drugs (e.g. opioids, cannabinoids, cocaine). Dependence on morphine was not included if associated with diseases of chronic pain
Parent diagnosed with ADHD	Diagnosed with ADHD in a psychiatric ward according to the Danish Psychiatric Nationwide Case Register
Parental unemployed > 21 weeks	Unemployment for at least one parent: The number of days unemployed (more than 21 weeks) during a calendar year. From registers of Income Compensation Benefits, Labor Market Research, and Unemployment Statistics. Parental unemployment for one or both parents
Parental violence	Battered adults according to hospitals admissions. Parent exposed to assault or injuries of undetermined intent. Victims of violence, which led to hospitalization and professional assessment that the injury was willfully inflicted by other persons. *Parent charged for violence:* The Criminal Statistic Register records persons charged for violence. This category comprises a wide range of criminal behavior of various degrees of seriousness: manslaughter, grievous bodily harm, violence, coercion and threats. This category does not include accidental manslaughter in combination with traffic accidents, or rape, which belongs to the category of sexual offences. The person has been charges and the decisions could be one of following: Guilty decisions, unsuspended/suspended, fine, withdrawal of charges. Other decisions could be: not guilty decisions, charges waived, or acquitted
Child maltreatment syndrome	Child being victim of violence, physical abuse, sexual abuse, psychological abuse, neglect or abandonment, which led to hospitalization and professional assessment of the injury being willfully inflicted by other persons (E960–E969, 796.00, 796.01, 796.02, according to the Danish modification of the ICD-8 classification; or T74, Z61, Y07.1, Y06.1, according to ICD-10 classification)

**Table A2:** Indicators of disintegrative family

***Risk factor:***	
Child in (public) Care	The child is living with the parents under caseworker supervision according to the Children’s Act, or the child is placed outside the home living in an institution or in a foster home. Information from the population based register of social assistance to children in care
Child adopted	Information about adoption is based on a combination of three administrative databases (1) the fertility database (2) the Central Population Register (CPR) and (3) the Medical Birth Register (MFR)
Family separation	Information on all children who had experienced divorce, separation and or the death of a parent before they were 18 years old, taken from the Danish Central Population Register (CPR) that connects children to their parents whether they are married or not (Christoffersen, 1995)

**Table A3:** Indicators of community violence

**Indicators**	**The school child:**
Disadvantaged Area	A governmental board has identified the most disadvantaged housing areas. These are a part of the subsidized housing sector, consisting of 135 areas. About 4% of the population (200,000 persons) lives in these areas. Each area has 1,500 inhabitants, on average, ranging from 30 to 14,000 persons (Boligministeriet, 1993; Graversen et al., 1997). These disadvantaged housing areas were divided into quintiles and the two most-disadvantaged quintiles were identified as disadvantaged areas by this dichotomized variable. These most disadvantaged areas would thus cover about 80,000 inhabitants or 1.6% of the total population
Charged of a violent crime	The child: Persons who have been confined with violent personal crimes under the Danish Penal Code e.g. offences against personal liberty, violence against the person, homicide, assault, robbery, but not theft (Christoffersen et al., 2003b). The person has been charges and the decisions could be one of following: Guilty decisions, unsuspended/suspended, fine, withdrawal of charges. Other decisions could be: not guilty decisions, charges waived, or acquitted
Victim of a sexual crime	The child: Persons who have been victim of sexual offence under the Danish Penal Code recorded by police. Including criminal reports for rape, sexual abuse, sexual exploitation, incest, indecent exposure, when the victim was younger than 18 years (Christoffersen, 2020)
Victim of a violent Crime	The child: Persons who have been victim of violent personal crimes under the Danish Penal Code e.g. offences against personal liberty, violence against the person, homicide, assault, robbery, but not theft (Christoffersen, 2019)

**Table A4:** Individual disabilities

**Disabilities:**	**Indicators of child’s vulnerabilities:** The World Health Organization (WHO) International Classification of Diseases (ICD-10)
Autistic spectrum disorder	Autism F84 Pervasive developmental disorders
Speech disability	Specific developmental disorders of speech and language ICD-10:F80, Speech disturbances ICD-10:R47, Lack of expected normal physiological development ICD-10:R62
ADHD	Diagnosed with ADHD in a psychiatric ward according to the Danish Psychiatric Nationwide Case Register. ADHD F90 Hyperkinetic disorders and/or receiving ADHD-drugs 'N06BA04' or 'N06BA09'
Loss of hearing	Conductive and sensorineural hearing loss ICD-10:H90-H91, Other disorders of ear ICD-10:H93-95
Epilepsy	Acquired aphasia with epilepsy ICD-10:F80.3, Epilepsy ICD-10:G40
Mental retardation	Mental retardation ICD-10:F70-F79
Down’s syndrome	Chromosomal abnormalities not elsewhere classified: ICD-10: Q90
Brain injury	Intracranial injury S06, Other mental disorders due to brain damage and dysfunction and to physical disease F06, Post-concussional syndrome ICD-10: F07.2, Chronic post-traumatic headache ICD-10: G44.3
Stuttering	Stuttering and other behavioral and emotional disorders with onset usually occurring in childhood and adolescence ICD-10:F98
Physical disabilities	Symptoms and sign involving the nervous and musculoskeletal systems ICD-10:R25-R29, Injuries of neck and trunk, limp, ICD-10:T91-T94
Dyslexia	Specific developmental disorders of scholastic skills ICD-10:F81. Dyslexia and other dysfunctions, not elsewhere classified ICD-10:R47
Blindness	Low vision ICD-10:H54
Cerebral Palsy	Infantile cerebral palsy ICD-10: G80,
Congenital malformations	Congenital malformations, deformations and chromosomal abnormalities ICD-10:Q00-99

Source: (Christoffersen, 2020)

**Table A5:** Demographic variables

**Indicators**	**The school child:**
Mother teenager	The mother had been a teenager herself when she gave birth to the child in focus
Danish/ non-Danish Citizenship	The definition is based on fulfilling one of the following conditions: (a) If at least one of the parents has Danish citizenship and is born in Denmark. (b) If there is no information in the registers about any of the parents and the child himself/herself has Danish citizenship and is born in Denmark. (c) All others are defined as non-Danish
Gender (boys = 1)	

**Table A6:** Indicators of previous psychosocial stressors, substance abuse

Indicators of Maltreatment	The school child:
Suicide attempt	The child: Self-inflicted harm according to hospitals admissions. The definition of suicide attempts also included behavior that conformed to the following conditions: (i) Suicide attempts that had led to hospitalization, (ii) assessment of the trauma being an act of self-mutilation according to the international statistical classification of injuries when discharged from hospital, (iii) the trauma had to be included in a specified list of traumas traditionally connected with suicide attempts: cutting in wrist (carpus), firearm wounds, hanging, self-poisoning with drugs, pesticide, cleaning fluids, alcohol or carbon monoxide. This does not include non-suicidal self-harm (Christoffersen et al., 2003a, b)
Drug abuse	The child: Addiction or poisoning by drugs according to hospitals admissions. Mental and behavioral disorder due to use of drugs (e.g. opioids, cannabinoids, cocaine). Dependence on morphine was not included if associated with diseases of chronic pain
Alcohol abuse	The child: According to hospital admissions the following diagnoses were expected to be associated with long-term alcohol abuse: Alcoholic psychosis, alcoholism, esophageal varices, cirrhosis of liver (alcoholic), chronic pancreatitis (alcoholic), delirium, accidental poisoning by alcohol. Mental and behavior disorder due to use of alcohol also included (Christoffersen & Soothill, 2003)
